# Spontaneous Giving under Structural Inequality: Intuition Promotes Cooperation in Asymmetric Social Dilemmas

**DOI:** 10.1371/journal.pone.0131562

**Published:** 2015-07-08

**Authors:** Sebastian Lotz

**Affiliations:** 1 Stanford University, Department of Sociology, Stanford, California, United States of America; 2 University of Lausanne, Department of Economics, Lausanne, Switzerland; Wenzhou University, CHINA

## Abstract

The present research investigates the role of intuitive mental processing on cooperation in experimental games involving structural inequality. Results from an experiment using conceptual priming to induce intuitive mental processing provide the first evidence that cooperation is promoted by intuition in an asymmetric context that distributes the gains from cooperation unequally among a group. Therefore, the results extend our understanding of the cognitive underpinnings of human cooperation by demonstrating the robustness of intuitive cooperation in games involving structural inequality regarding asymmetric gains from cooperation. Additionally, the results provide the first successful conceptual replication of the intuition-cooperation link using conceptual priming, therefore also contributing to the debate about the validity of previous research in other contexts. Taken together, the present research contributes to the literature on psychological and institutional mechanisms that promote cooperation.

## Introduction

One of the most widely investigated phenomena in human social life is why and how cooperation can be sustained despite individual incentives to free-ride [1, 2]. Research has identified many mechanisms that foster cooperation, among them direct and indirect reciprocity [3, 4], spatial and multilevel selection [5, 6] as well as kin selection [7]. Yet, even in the absence of such mechanisms, for instance in anonymous one-shot interaction over the internet, we observe cooperation rates far above the theoretical predictions [8]. But why is that?

One explanation is that intuitions and norms that guide our real-world behavior carry over to such artificial laboratory situations and trigger an evolutionary optimal response rather than a game-theoretic optimum for this specific context. While the general mechanism of how this happens is debated ([9] for an overview], the fact that cooperation exists in these situations is quite robust. In this respect, ample amount of research supports the hypothesis that intuition promotes cooperation and reciprocity, implicating that cooperation is a “system 1” process. And in fact, theoretically grounded in dual-process theories of judgment and decision-making [10–14], several researchers reported positive effects of intuition on cooperation in social dilemmas as well as pro-sociality in general throughout several studies and thousands of observations.

For instance, the induction of an intuitive mindset through priming leads to increased cooperation in simple economic games [15] and promoting intuition by means of cognitive-load increases generosity in resource allocation [16] as well in simple distribution tasks such as the dictator game [17, 18]. Furthermore, people’s affective, emotional responses have been linked to various domains of pro-social decision making, such as gratitude to pro-social behavior [19] or altruistic interventions [20, 21].

But interestingly, the impact of intuition on cooperation has uniquely been measured in symmetric contexts, meaning that costs and benefits are equally distributed among the participants. Yet, in real life, this is hardly ever the case. For instance, when cooperating with one’s boss, it is quite plausible that he or she takes a larger portion of the gains from cooperation. If you are remodeling your house, it might well be that one spouse likes the outcome much more than the other. And in artificial social dilemma games, the gains from cooperation might also be biased to benefit one party more than the other without changing the inherent characteristics of the game (i.e., that it is individually optimal to free-ride and socially optimal to contribute). And in fact, previous research has addressed the question of heterogeneity in initial endowments [22–24], marginal per capita returns [25, 26], and different perceptions in fairness norms [27, 28] regarding public goods. In addition, heterogeneity was investigated by introducing leadership [29–33], which had positive results on cooperation. However, the general results regarding heterogeneity seem diverging and Ledyard [34] points out that the effect of heterogeneity on cooperation is quite weak: “There does not yet appear to be enough evidence for acceptance. In many cases there is conflicting evidence. (p. 158)”. This particularly applies to the present context in which the differences in marginal returns are rather small and the interaction is one-shot (see [34] for a detailed discussion).

But what does intuition do in these cases of structural inequality? Given previous experimental results that suggest that people cooperate more if deciding intuitively [15] and other results that highlight a higher sensitivity towards equality under the same cognitive process [17, 18, 35], intuition could have positive or negative effects on cooperation. The present research is directly designed to test this question. Thus, the research is exploratory in the sense that there are conflicting hypotheses based on existing research. If intuition favors cooperation [15], then structural inequality should be irrelevant for the positive effect of intuition on cooperation. However, if intuition favors equality concerns rather than cooperativeness, as has been suggested in various experiments, then intuition could even be detrimental for cooperation in such situations that involve inequality. Therefore, the present research is designed to explore these two potential outcomes by disentangling the cooperative element from equality.

Besides answering this important question and despite the overwhelming support of intuitive processes on pro-social decision making, especially the results reported in Rand et al. [15] triggered a lot of attention in a debate around the replicability and generalizability of the intuition-cooperation link. Although not the main focus of the present research, it also contributes to the debate about whether or not intuition promotes cooperation. The conclusion that people are intuitively cooperative in social dilemma games was challenged by several researchers [36, 37]. Interestingly, none of these replication attempts relied on conceptual priming, but used decision-times or experimental inductions of time-pressure to measure or incur intuitive decision making. However, especially the effect of time pressure on cooperation has repeatedly been shown [38–41]. Therefore, it is generally assumed that time-pressure puts people into an intuitive mode, causing intuitive decision making [15].

Importantly, recent research qualified results on decision-time correlation vs. time pressure manipulation by showing that decision time correlates with extreme responses while actually experimentally manipulating decision times using time pressure increases cooperation and decreases extreme selfishness. Correlational findings can therefore be explained by decision conflict rather than by degree of intuitiveness [42]. However, there is still limited support by exact nor conceptual replications that experimentally inducing intuition by alternative means leads to increased cooperation. Addressing this issue in asymmetric games, therefore, provides a viable avenue for research on the intuition-cooperation link.

Noteworthy, the present research does not make exact replications of the intuition-cooperation link, and especially a replication of Study 8 reported in Rand et al. [15], obsolete. As many scholars have noted [43–47], direct replications serve to investigate the reproducibility of a certain effect, whereas conceptual replication serves as a validation of a previously established effect. The research question here was whether or not intuition promotes cooperation in asymmetric contexts (i.e., as this has not been investigated before and is interesting due to the ability to disentangle cooperativeness from equality), thus a direct replication of Rand et al.’s Study 8 [15] would be irrelevant to the present context that involves asymmetry, despite being an interesting (and relevant) research question in itself.

Summarizing, the present research is designed to test the impact of intuitive mental processing on cooperation in a social dilemma game involving asymmetric payoffs to the participating parties. While this analysis presents a gap in the literature itself and is the main focus of the research, the experiment also contributes to the debate about the previously established relationship that intuition favors cooperation [15] and its validation within a different context.

## Materials and Method

Following recent sample size suggestions [47], a total of 250 HITs were created on Amazon Mechanical Turk ([48], for validity of AMT as a research tool), with a stopping rule automatically enforced upon completion of these HITs. The HIT was named “Decision-making task” and the description of the task was as follows: “Work on a short task related to decision making (max. 10–20 minutes), up to $1.00 bonus”. The keywords were “psychology questionnaire” and “decision making”. Furthermore, the HIT required an approval rate for all requesters’ HITs greater than or equal 95 and the sample was restricted to locations in the US. There were no time limits in either part of the experiment, but the maximum time for completion was 60 minutes. This limit did not time out any participant.

The study received approval by the institutional review board (Stanford University IRB Panel on non-medical human subjects: No: 349, Panel 2, Protocol ID: 30556) and all participants gave their informed consent by checking the corresponding box before taking the study. Out of the 250 participants, a total of 4 participants aborted the experiment before the assessment of the dependent variable, leaving 246 for analysis. Participants (60% females) were on average 29.37 years (SD = 8.65, ranging from 18–61). Cognitive processing was manipulated using a conceptual prime that was well-established and used in previous research [49]. This research has demonstrated the power of these specific primes to promote intuitive versus reflective thinking in the domain of religious belief, and these findings were validated in a subsequent study using a different method [50]. The present research used the identical prime used in Rand et al.’s Study 8 [15, 49]. Prior to the measurement of the main dependent variable, participants were asked to write about a time in their life where intuition worked out well, or reflection worked out poorly (both promoting intuition); or the opposites (both promoting reflection). Thus, while manipulating cognitive style, the procedure also counterbalanced valence, to observe (and control for) potential effects of valence on cooperation, for which previous research could not establish a clear relationship [51–53].

Subsequently, participants proceeded to a 4-person Public Goods Game (PGG), with the form of

$$\pi_{ij}\;=\;X_{i}-C_{i}+{2\;*\beta }_{j}\sum_{k\;=\;1}^{4}{Ci}_{k}$$ (1)

in which *π*_*ij*_ denotes the payoff of the individual player *i* of type *j*, *X*_*i*_ denotes the initial endowment (40 cents), *C*_*i*_ the individual contribution (0 ≤ *C*_*i*_ ≤ *X*_*i*_), and *β* the marginal per capital return with *β* < 0.5 for all types *j*, ensuring a dilemma at the individual level.

Structural inequality was manipulated by having participants play the PGG in one of two roles *j*, therefore manipulating their β. Two ‘low opportunity’ players each receive β = 0.2 (i.e., whose *MPCR* is, therefore, 0.4), while two ‘high opportunity’ players each receive β = 0.3 (i.e., whose *MPCR* is, therefore, 0.6). Thus, cooperation always led to inequality because participants profited differently from the group project. After assessment of behavior, participants were asked to answer a comprehension check to ensure that they have correctly understood the nature of the social dilemma game (i.e., the critical issue that it is individually optimal to free-ride, yet socially optimal to contribute everything to the public good). These questions were: “What is best for you individually?” and “What is best for the group?” Answering options were: invest everything vs. keep everything.

In addition to $0.30 base pay, all participants received $0.40 that they could invest in the group project. Each cent invested into the group project was doubled. After the decision, further demographics were assessed besides comprehension checks (see S1 and S2 Tables for detailed experimental instructions).

## Results and Discussion

**Fig 1:**
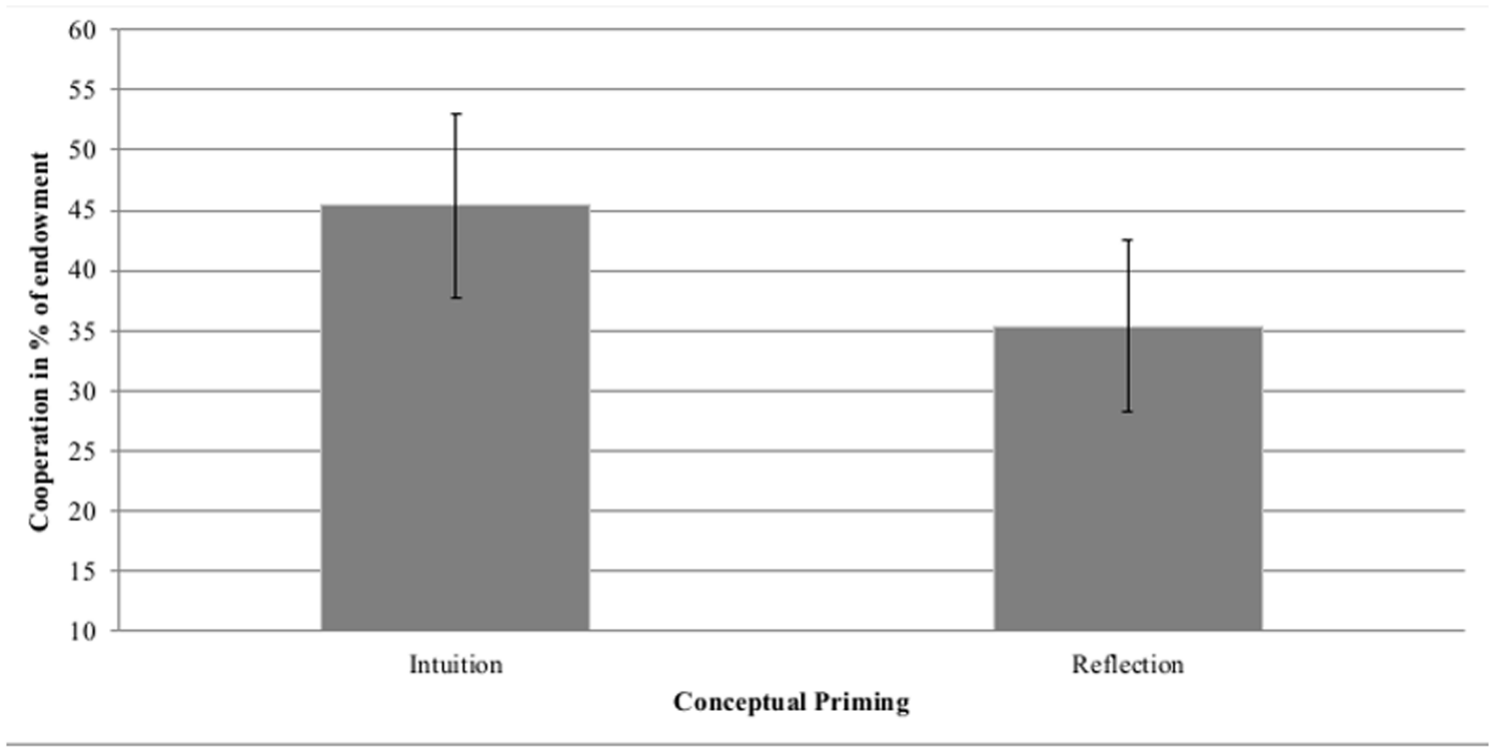
Mean Rates of Cooperation. Cooperation is displayed by conceptual priming (Intuition: intuition good/reflection bad vs. Reflection: intuition bad/ reflection good). Error bars represent 95%-level confidence intervals. Intuition refers to primes that promote intuition or inhibit reflection (intuition good, reflection bad), reflection refers to primes that promote reflection or inhibit intuition (reflection good, intuition-bad). Based on *n* = 246 observations.

The results support the stream of literature that shows that intuition increases cooperation. In total, the results show an overall positive effect of intuition on cooperative decision-making in the one-shot asymmetric social dilemma (for an overview of descriptive results, see Table 1, see Fig 1 for mean cooperation rates by priming condition). Importantly, no variable differed as a function of the manipulation except cooperation (i.e., the conditions did not over-represent any age groups or gender and the manipulation had no impact on comprehension or the length of the written paragraph).

**Table 1:** Descriptive Results.

	Intuition-Bad (n = 66)		Reflection-Good (n = 61)		Reflection-Bad (n = 57)		Intuition-Good (n = 62)	
	Mean	Std.	Mean	Std.	Mean	Std.	Mean	Std.
Contribution (in %)	34.96	41.08	35.82	40.69	50.48	43.51	40.81	40.60
Age	29.56	7.54	30.02	9.70	30.37	10.18	27.79	7.01
Gender (0 = M, 1 = F)	0.47		0.36		0.39		0.38	
Comprehension I	.89	.31	.90	.30	.86	.35	.97	.18
Comprehension II	.98	.17	.97	.18	.98	.13	.92	.28
Paragraph length	785	356	782	248	811	268	759	282

*Notes*: All respondents were US-based, comprehension I (“What is best for you individually?”), comprehension 2 addresses group level (“What is best for the group?”). There are no statistical differences of age, gender, comprehension checks, and paragraph length with respect to manipulations. Paragraph length is indicated by characters in the statement.

In order to estimate the effect of intuition on cooperation, Tobit regressions were used to account for the fact that subjects may contribute nothing or their entire endowments. Throughout all models, robust standard errors were calculated. The experimental manipulations were dummy-coded (e.g., intuition = 1 if an intuitive mindset was induced, and 0 otherwise; or valence = 1 if positive, and 0 otherwise). Table 2 displays the regression models. Model 1 shows that intuition positively affects cooperation (p = 0.034) in the absence of any controls, while valence (p = 0.707) as well as the interaction (p = 0.199) do not significantly predict cooperation. Model 2 controls for age, gender, comprehension, as well as the length of the written paragraph and corroborates the initial findings: The effect of intuition remains significant (p = 0.035).

**Table 2:** Results from Tobit Regressions.

	(1)	(2)	(3)	(4)
**Intuition**	46.4771** (2.13; 21.83)	48.9130** (2.35; 21.76)	46.4098** (2.12; 21.89)	49.2492* (1.82; 27.02)
**Valence**	7.6360 (0.38; 20.29)	6.3109 (0.32; 19.45)	7.6442 (0.38; 20.29)	7.6890 (0.38; 20.29)
**Mar. Return**			0.4577 (0.03; 14.69)	2.9836 (0.15; 20.29)
**Intuition x Valence**	-37.7958 (1.29; 29.32)	-37.9254 (-1.32; 28.83)	-37.7073 (-1.28; 29.49)	-38.3261 (-1.29; 29.68)
**Intuition x Mar. Return**				-5.2638 (-0.18; 29.41)
**Age**		1.56914* (1.71; 0.92)		
**Gender**		8.5931 (0.60; 14.42)		
**Compreh**.		-66.5323***(-3.32; 20.03)		
**Paragraph length**		0.0415 (0.02; 1.66)		
**Constant**	9.7746 (0.65, 15.07)	-26.5195 (-0.58; 45.42)	9.5625 (0.58; 16.63)	8.3932 (0.47; 17.94)
**N**	246	246	246	246
**Pseudo R-squared**	0.004	0.019	0.004	0.004

*Notes*: Numbers present unstandardized regression coefficients, t-values are presented in parentheses before robust standard errors,

*p<0.10,

**p<0.05,

***p < .001, dependent variable: cooperation in percentage, coding: Intuition, valence, marginal return were dummy-coded (1 = intuition, positive, high/0 = reflection, negative, low), age (continuous), gender (1 = Female, 0 = Male), comprehension (combined measure from two items: 1 = yes, 0 = no), paragraph length (continuous, number of characters).

Next, the impact of the *MPRC* was tested. Using a simple t-test, cooperation did not differ depending on the marginal return (p = 0.710). In Tobit regressions, *MPCR* was also dummy-coded to represent high (1) or low (0) opportunity players. First of all and consistent with reviews on heterogeneity in public goods [34], no main effect of *MPCR* on cooperation was observed in this one-shot game (p = 0.748, Model 3). Furthermore, a Tobit regression assessed the main effect as well as the interaction effect of the intuition prime and *MPCR* (high vs. low *MPCR*). There is no significant interaction between promoting intuition and *MPCR* (see Model 4). Controlling for age, gender, or paragraph length does not change the main result that intuition favors cooperation. Additionally controlling for comprehension in various models that involve a combination of none, some, or all control variables produces result that are at least marginal significant (all p’s < 0.10)

Summarizing, besides the conceptual replication of previous results [15], the present results supports the positive relationship of intuition and cooperation. While intuition promotes cooperation even in a game with structural inequality, no interaction occurred between the manipulation and the role that the participant decided in.

## Conclusion

The present research showed that intuitive mental processing—induced by conceptual priming—promotes cooperation even in a social dilemma that does not allocate the gains from cooperation equally among the participants. Thus the results advance our understanding of the cognitive underpinnings of cooperation in games where participants benefit unequally from contributions to the public good. Many real-life decisions involve asymmetric gains from joint cooperation, yet the effect of intuition on these types of dilemmas has thus far been neglected in research. The present research was designed to fill that gap in the literature. The results deserve some discussion and the intuition-cooperation link deserves much more research attention.

First, the results provide the first successful conceptual replication from an independent research lab of the highly debated results presented in Rand et al. [15], using the conceptual priming technique [49] rather than the uniquely utilized time-pressure manipulation [36, 37]. By means of conceptual replication, the results therefore validate the conceptual priming finding by showing that it extends to social dilemmas involving structural inequality. Naturally, this conceptual replication does not render exact replications useless [43–47]. For instance, future research could investigate whether expertise effects ([15], Study 9) also moderate the effect of intuition on cooperation under structural inequality. In addition, a large-scale replication effort involving many independent labs should attempt replication of the basic effects reported in Rand et al.’s [15] studies that use conceptual priming to induce an intuitive mindset.

Second, this novel evidence presented here suggests that intuition favors cooperation when equality concerns would lead to non-cooperation. While various research might suggest that intuition may activate equality concerns that may have detrimental effects on cooperation under structural inequality, the results show that intuitive cooperation overrules fairness concerns in asymmetric contexts. The fact that promoting intuition nonetheless increases contributions suggests that intuition favors efficiency more strongly than equality, or perhaps a more general tendency to cooperate [54]. In other words, the fact that cooperation gains are equally distributed is not the underlying cause of the intuition-cooperation link. Importantly, the present context involved two players with high *MPCR*. Cooperation between the two already paid off. However, in the symmetric case, cooperation between two is also sufficient to avoid losses from cooperation. Future research could investigate if two different types of players in a group facilitates cooperation, for instance by reducing perceived uncertainty about others behavior. This effect could potentially be independent of *MPCR* and one way to investigate this hypothesis is to give the two types different names (e.g., *red* and *blue* players).

Third, future research can address whether the specific framing of the study (e.g., by means of instructions) affect the outcome in this game. For instance, the present research also relied on rather vivid instructions (e.g., “Each cent invested in doubled.”) Possibly, this vivid framing might be associated with triggering cooperative responses, especially if in an intuitive mindset. The present research relied on instructions that mirrored Rand et al.’s [15] instruction as much as possible, but future research could systematically vary the vividness of the instructions, for example by stating the *MPCR* rather than the phrase to “double each cent”.

Fourth, this research also shows that replication attempts should involve several manipulations before dismissing previously published research results, especially if these studies used several methods to show the effect initially. As Rand et al.’s [15, 55] initial result has been challenged by several null findings relying on the same induction of intuition [36, 37], none of the research assessed the effect with a multi-method approach before dismissing it as non-replicable or invalid in other contexts. The results reported here suggest that process-oriented manipulations (e.g., priming) can be more effective in activating the psychological concept of interest. Importantly, the effect sizes (R^2^’s) in the present research were quite small. Thus, future research could also address the sizes of certain effects and compare how different tools to promote cooperation perform relatively. This is particularly important when it comes to using psychological principles outside the lab (e.g., in domains around behavioral science and policy, marketing, organizational behavior, etc.).

Finally, slightly changing the game to involve unequal players may be enough to overcome expertise effects, similar to changing the framing of the game [38]. Research has shown that subjects recruited on Amazon Mechanical Turk may become too experienced to be receptive to subtle effects of primes ([15], Fig 3a therein). Ideally, this would be tested using a design which manipulates structural inequality, intuitive vs. reflective decision making, and measures subjects’ experience. To sum up, the present research provided further results on the intuition-cooperation link in a context that involves structural inequality, implying that symmetric gains are not a pre-requisite for intuitive cooperation.

## Supporting Information

S1 Dataset Dataset. (XLSX)

S1 Table Instructions for the Priming Task. (DOCX)

S2 Table Instructions for the Public Goods Game under Structural Inequality. (DOCX)
