# Supplementary material for: Spontaneous Giving under Structural Inequality: Intuition Promotes Cooperation in Asymmetric Social Dilemmas
Source: PLoS One. 2015 Jul 8;10(7):e0131562. doi: 10.1371/journal.pone.0131562 (PMC4496099; doi:10.1371/journal.pone.0131562)
Supplement: S1 Table — (DOCX) [file pone.0131562.s002.docx]

**S1 Table. Instructions for the priming task**

| *Intuition-bad* | „Please write a paragraph (approx. 8-10 sentences) describing a time your intuition/first thought led you in the wrong direction and resulted in a bad outcome.” |
| --- | --- |
| *Reflection-bad* | „Please write a paragraph (approx. 8-10 sentences) describing a time carefully reasoning through a situation led you in the wrong direction and resulted in a bad outcome.” |
| *Intuition-good* | „Please write a paragraph (approx. 8-10 sentences) describing a time your intuition/first thought led you in the right direction and resulted in a good outcome.” |
| *Intuition-good* | „Please write a paragraph (approx. 8-10 sentences) describing a time careful reasoning through a situation led you in the right direction and resulted in a good outcome.” |
