# Supplementary material for: Spontaneous Giving under Structural Inequality: Intuition Promotes Cooperation in Asymmetric Social Dilemmas
Source: PLoS One. 2015 Jul 8;10(7):e0131562. doi: 10.1371/journal.pone.0131562 (PMC4496099; doi:10.1371/journal.pone.0131562)
Supplement: S2 Table — (DOCX) [file pone.0131562.s003.docx]

**S2 Table. Instructions for the Public Goods Game under structural inequality**

| You have been randomly assigned to interact with 3 other people. All of you receive this same set of instructions. You cannot participate in this study more than once.  Each person in your group is given 40 cents for this interaction (in addition to the 30 cents you received already for participating). You each decide how much of your 40 cents to keep for yourself, and how much (if any) to contribute to the group’s common project (in increments of 2 units: 0, 2, 4, 6 etc.). All money contributed to the common project is doubled, and then split among the 4 group members. There are two types of persons (A and B), and both types receive different amounts out of the group’s common project after the interaction.  If you represent one of the two Type A persons in your group, you will receive 30% of the money in the common group project. Thus, a Type A person receives 1.2 cents back for every 2 cent he/she invests and provides a total of 2.8 cents to the three others.  If you represent one of two Type B persons in your group, you will receive 20% of the money in the common group project. Thus, a Type B person receives 0.8 cents back for every 2 cents he/she invests and provides a total of 3.2 cents to the others.  If everyone contributes all of their 40 cents, everyone’s money will increase: Type A persons will earn 96 cents and Type B persons will earn 64 cents.  But if everyone else contributes their 40 cents while you keep your money, you will earn more (and the others will earn less). That is because for every 2 cents you contribute, you get less than 2 cents back. Thus you personally lose money on contributing, but the others gain more than you lose.  The other people are REAL and will really make a decision – there is no deception in this study. Once you and the other people have chosen how much to contribute, the interaction is over. Neither you nor the other people receive any bonus other than what comes out of this interaction.  You will learn on the next screen whether you represent a Type A player or a Type B player. |
| --- |
